# Peer Effects in Unethical Behavior: Standing or Reputation?

**DOI:** 10.1371/journal.pone.0122305

**Published:** 2015-04-08

**Authors:** David Pascual-Ezama, Derek Dunfield, Beatriz Gil-Gómez de Liaño, Drazen Prelec

**Affiliations:** 1 Department of Financial Economy and Accounting II, Universidad Complutense de Madrid, Madrid, Spain; 2 Sloan School of Management, Massachusetts Institute of Technology (MIT), Cambridge, MA, United States of America; 3 Departament of Methodology and Social Psychology, Universidad Autónoma de Madrid, Madrid, Spain; 4 Sloan School of Management, Massachusetts Institute of Technology (MIT), Cambridge, MA, United States of America; University of the Basque Country, SPAIN

## Abstract

Recent empirical evidence shows that working in an unsupervised, isolated situation under competition, can increase dishonest behavior to achieve prestige. However, could working in a common space, in the presence of colleagues affect cheating? Here, we examine how familiar-peer influence, supervision and social incentives affect worker performance and dishonest behavior. First, we show that working in the presence of peers is an effective mechanism to constrain honest/dishonest behavior compared to an isolated work situation (experiment 1). Second, we demonstrate that the mere suspicion of dishonesty from another peer is not enough to affect individual cheating behavior (experiment 2), suggesting that reputation holds great importance in a worker’s self-image acting as a strong social incentives. Third, we show that when the suspicion of dishonesty increases with multiple peers behaving dishonestly, the desire to increase standing is sufficient to nudge individuals’ behavior back to cheating at the same levels as isolated situations (experiment 3).

## Introduction

Imagine you are alone at an empty crosswalk and the light is red. Most of us would simply cross the street. If we arrive at the crosswalk and find a group patiently waiting, however, we are likely to follow the norm: stop and wait. If a single person breaks the norm and crosses, the temptation to jaywalk increases. Still, the action of the group may hold sway, so we would likely wait for the light, but if more than one person decides to jaywalk the peer pressure is enough that the whole group would cross as well. As this example shows, peer effects can be very important in our daily life, changing our “natural” behavior compared to when we are alone. This is especially true when there is a possibility to act dishonestly or contrary to social norms because of a lack of supervision or punishment. If we are tempted to act dishonesty, peer effects could suppress our intention, but along the same line, unethical peer behavior could also encourage us to act dishonestly.

In a labor market context, ethical/unethical behavior can be influenced by (at least) three possible causes: 1. the influence of others (peer effects), 2. incentives, and 3. supervision. Regarding *peer effects*, peers´ unethical behavior can influence an observer’s behavior in different ways [1]. When exposed to the dishonesty of others, individuals may change their estimate of the likelihood of being caught cheating, increasing one’s propensity to act dishonestly [2]. Alternatively, the dishonesty of others may affect the saliency of ethicality at the decision-point of cheating, decreasing one’s propensity to act dishonestly [3]. Also, observing the unethically of another person may simply change one’s understanding of the social norms related to dishonesty [4]. The social-norms mechanism suggests that when the observed “other” is an out-group member, in-group members will show a reduced likelihood of engaging in dishonest behavior. When the observed “other” is an in-group member, however, social norms for the group could change, and an individual may be more likely to engage in dishonest behavior [1]. There is wide literature about peer effects of out-group members in cheating, so we will focus our work in “in-group peer effects.” In-group effects are of particular interest in organization behavior because we assume that everyone who works frequently in the same place belongs to the same peer group [5]. In a labor market context, we can find two types of peers (familiar and non-familiar), each with three levels (subordinates, workmates, and supervisors). The difference between familiar peers and non-familiar peers is based on daily personal relationships. We define Familiar-Peers (FP) in a labor market context as the people who work in an individual’s closer circles, daily, over a long period of time (not occasionally), and who hold a close, personal relationship (e.g. have lunch together regularly, talk about family, etc.). Non Familiar-Peers are the rest of the people in the organization who have a non-daily, basic labor relationship.

That classification is very important when we analyze a second influencer of ethical/unethical behavior in the workplace: *incentives*. Employees are “rational cheaters” [6] and firms can anticipate and respond to the consequences of their unethical behavior with incentive systems. According to Ariely et al. [7], the expectation that increasing performance-contingent incentives will improve performance rests on two subsidiary assumptions: (1) that increasing performance-contingent incentives will lead to greater motivation and effort and (2) that this increment in motivation and effort will result in improved performance. In many cases, these assumptions hold true, however, research has also shown that economic incentives that are either too low or too high can actually decrease performance [7,8]. Incentives that are too high can also increase dishonest behavior [9]. Along the same line, recent empirical evidence shows that social incentives, such as prestige or standing, increase efficiency and therefore improve performance, but, like large economic incentives also increase dishonest behavior [10]. Under competition, individuals increase their effort [11], but when given the opportunity to cheat that effort is muted [12].

Finally, a third influencer on ethical/unethical behavior is *supervision*. Supervision is one of the most important mechanisms that a firm can use to avoid dishonest behavior. In organizations it can be either direct (carried out by organizations) or indirect (carried out by peers); however, it is very expensive and the relationship between supervision and performance is not well understood. Many researchers have tried to understand the role of direct supervision on performance by studying other variables like motivation or effort without clear results. Falk & Kosfeld [13], have suggested that close supervision of workers can undermine intrinsic motivation and decrease performance, while other researchers, such as Ariely, Kamenica & Prelec [14], have proposed that the way in which monitoring is framed crucially influences its effect on motivation and performance. Moreover, this link is more difficult to understand if we take into account that employees in the labor market have many incentives for being dishonest, whether they are ordinary workers or top executives [9]. On the other hand, indirect supervision by peers has been poorly studied as an alternative or complementary method to organizational direct supervision. According to rational crime theory, working in the presence of others increases the likelihood of being caught compared to working in isolation. This increased risk can then change workers cost-benefit calculation for cheating [2]. In many cases, especially in small business, indirect supervision may be the only type of affordable supervision. This type of supervision has been shown to break down if a peer is dishonest. Gino et al. [1] found that when a confederate cheated ostentatiously by finishing a task impossibly quickly and leaving the room with the maximum reward, his or her peers’ level of unethical behavior increased. However, in a workplace setting, it is unusual for someone to observe dishonest behavior directly. Instead, workers may simply suspect of dishonest activity. Moreover, the level of suspicion and the effect on individual behavior may depend on the type of peer and the social context. It is unknown if these less clear signals are sufficient to promote unethical behavior due to “one bad apple.”

The objective of this paper is to offer a deeper understanding about the spillover of peer effects, social incentives, and supervision on workers’ performance and cheating. These concepts may be quite closely related, all playing a large role modulating (dis)honest behavior in labor markets. One of the problems associated with peer effects research is the difficulty to separate a group’s influence on individual behavior from an individual’s influence on the group [15]. This problem is typically resolved by studying situations in which a “natural experiment” occurs, or in which individuals are randomly assigned to peers groups [16–20]. Our experiments conform to both of these solutions, with students related to their peers groups in a similar fashion to the labor market (a natural experiment) and with students randomly assigned to peer groups by the university admission process. We also provide a different point of view from previous literature in several aspects. Firstly, with few exceptions [17] peer effects have been studied within anonymous and temporary peer groupings. In the present work, peer effects are analyzed in a pre-existing natural group, in which individuals were familiar with each other, and in which every participant will have to spend many hours with the rest of the group in the future. We have just called that Familiar-Peer which is directly related to endogenous peer effects described by Manski [21]. Secondly, previous research has been focused on behavioral changes when participants were confident that one or more of their peers were indeed cheating. In our study, participants could only suspect that peers may be cheating but were unable to confirm their suspicions, a situation that is much more ecologically, valid and common in the “real-world”. As such, we also analyze if suspicion of dishonesty is sufficient to change individual behavior. We hypothesize that, in a non/low supervision situation, the mere presence of familiar-peers who might observe your behavior (e.g. working in an office with your peers) will decrease the level of cheating compared to an isolated situation (e.g. working at home or in an individual office) (Exp. 1). Finally, we assess how suspicion of cheating by familiar-peers interacts with the effects of supervision when social incentives are given to the group. We will study how *standing* and *reputation*, as a form of social incentives, may interact with supervision in Familiar-Peer/Isolated situations. As terms like standing, status, prestige, image or reputation have been used interchangeably in the literature depending on the discipline (e.g. sociology, economics, marketing, etc), it has been very difficult to assess their effects conceptually and empirically (see [22–13] for a thorough review). We define *standing* as “*…the organization´s ranking on relevant criteria*, *which*, *as a whole (beyond their aggregate sum) form the relative position of that organization in the eyes of given constituencies*” [23]. *Standing* takes into account opinions from all of the organization, both familiar and non-familiar peers. On the other hand, we define *Reputation* as familiar peers’ opinions on general criteria based on a longitudinal perspective that goes beyond a particular period of time. That means that a worker with good standing in the organization can have a bad reputation in his or her closer circles or vice versa. For example, one can imagine this occurring when the procedure or the style to increase standing within a company includes exploiting subordinates or lying to supervisors. Since reputation may be more important than standing for familiar-peers, we hypothesize that under non/low supervision, cheating under pressure to achieve standing will only increase if the suspicion of dishonest behavior of other peers’ is very high (Exp 2&3).

### Experiment 1: Isolation versus Familiar-Peer Task

***“Alone at an empty crosswalk*, *most of us would cross the street*, *even when the light is red*. *If we arrive at the crosswalk and find a group waiting patiently*, *however*, *we are likely to follow the norm*, *stop and wait*.*”***

## Method

### Participants

A total of 133 Spanish undergraduate students (64% male; *M*_*age*_ = 19, *SD* = 1.22) participated in the study for approximately 3€. The study took less than 30 minutes and was conducted with paper and pencil.

### Design and Procedure

The experimental design was a 3x2 factorial study. Participants were randomly assigned to one of six conditions generated by crossing the level of Supervision [High Supervision (HS), Low Supervision (LS) and No Supervision (NS)] with the Presence of Others during the execution of the task: Isolated (I: N = 20, 20, 20) or accompanied by Familiar-Peers (FP: N = 24, 26, 23). Both independent variables were between subjects. Based on Pascual-Ezama et al. [10] we asked participants to complete a notebook of a popular word search “mind game” puzzles taken from a local newspaper. Subjects were initially given the notebook and told that they would be paid 0.55€ for finding 10 different words in each word search matrix. Having completed the first page, they were then asked whether they would be willing to complete a second page for 0.50€ (5¢ less). The process continued, with wages declining by 5¢ per sheet, until the subject decided to stop working. Participants had to complete at least 4 pages and there was no time limit. In I, each subject participated in the experiment alone, without the presence of other subjects in the lab. In FP, participants did the task at the same time, in the same room, but individually. In both I and FP situations, subjects were unaware of the other conditions.

For the HS condition, participants had to identify themselves by writing their names in the notebook and give it back to the researcher directly. In the LS condition, participants had to deposit their notebooks in a big stack of similar notebooks. No written identification was demanded. At the end of the session, for both conditions, participants would then report their result to receive the reward. To compare the real number of pages completed in the LS condition with those reported we manipulated the number of sheets of the notebooks in the stack: all the notebooks in the stack had 11 pages while participants’ notebooks had 12 pages. So, whenever a participant finished, the researcher could know which notebook was his or hers. To maintain confidentiality, researchers assigned a number to the participant and noted it on their notebook. Finally, in the NS condition, participants did not have to give the notebook to the researcher (they could shred it or take it away with them). NS participants then only had to report their result to the researcher to receive their reward. Hence, in the NS condition, we had no information about the real number of pages completed; only the number of pages declared. The experimental design allowed participants in the NS condition to be 100% confident they would not be caught cheating; participants in the LS condition, however, could be confident but not 100% sure. In all cases, we registered the number of pages reported (‘units declared’), the number of pages actually completed (only for HS and LS), and the time spent for each participant to complete the entire task. In order to have a measure of cheating, we calculated the number of units declared minus the number of units completed (*cheating* = *units declared—units actually completed)* for HS and LS conditions (as information for units completed in the NS condition was not available). This analysis allows us to determine the level of cheating in the HS and LS conditions. The bigger the difference is between the measures, the bigger the cheating is in a given condition. In NS, we used time as a proxy to measure cheating by comparing time spent under HS and LS conditions (see [10]).

Finally, participants were also offered a social incentive (in this and all the following experiments). Experiments were run during a regular semester undergraduate course as part of laboratory classes, and participants were told that the most efficient participants would be announced in class. The students voluntarily participated in the experiment, and the results did not affect their grade at the end of the semester. Therefore, after the experiment was completed, we explained it, showed the results, and (as promised), announced who had the best performance in each experiment. Taking into account that participants had to finish a minimum of 4 units, instructions defined efficiency as minutes spent by units declared, independently of the final number of units declared. That means that one participant who reported 4 units in 12 minutes (3 minutes per unit) was considered as more efficient than another participant who reported 10 units in 40 minutes (4 minutes per unit).

## Results and Discussion

### Performance

Mean number of units declared for all conditions are reported in S1 Table. A 3x2 ANOVA was run with Supervision (HS LS and NS) and Presence of Others (I and FP) as between subjects factors on units declared. Only a main effect of Supervision was found [F(2,127) = 3.67; p = .028; η^2^ = .06]. Differences appeared between HS (M = 9.24) and NS (M = 8.33) conditions (p = .027). Neither the effect of the Presence of Others nor the Interaction were significant (F<1, for both).

### Cheating

Cheating measures, “*cheating = units declared—units actually completed*,*”* for each condition can be seen in Table 1. We run a 2x2 ANOVA again with Presence of Others (I and FP) and Supervision (HS and LS, as no information is available for units done in NS. We found main effects of Supervision [F(1,86) = 191; p <.001; η^2^ = .69], Presence of Others [F(1,86) = 18.06; p = <.001; η^2^ = .17], and Interaction [F(1,86) = 21.72; p <. 001; η^2^ = .20]. There is a significant difference between HS and LS both for I and FP (p<.001, for both); meaning that dishonest behavior appears in both the Isolated and the Familiar Peer situations (see Table 1). However, importantly, in a planned comparison we observed a significantly larger cheating effect [t(86) = 4.7; p<.001] in I (*Mean* Difference = 4.5) than in FP (*Mean* Difference = 2.2). That is, although dishonest behavior is present under both situations, it is significantly reduced when Familiar-Peers are present compared to Isolated situations. On the other hand, although a main effect of the Presence of Others was significant, there are no differences between FP and I for HS (p = .77), while they appear for LS (p<.001). Those results show that Familiar-Peer effects are only found under Low Supervision but not under High Supervision.

**Table 1:** Units Declared, Units Actually Completed, Cheating, Honest People and Time per Unit through all the experiments.

Presence of Others	Supervision	Units Declared (UD)	Units Actually Completed (UAC)	Cheating (UD-UAC)	Percentage of Honest People	Time/Unit
I (Individual)	High Supervision (N = 20)	9.15	9.15	0	N/A	3.23
	Low Supervisión (N = 20)	8.75	4.55	4.20 (48%)	0%	2.13
	No Supervision (N = 20)	8.45	¿?	N/A	¿?	2.13
FP (Familiar Peer)	High Supervision (N = 24)	9.33	9.33	0	N/A	3.44
	Low Supervisión (N = 26)	8.50	6.27	2.23 (26%)	26%	3.44
	No Supervision (N = 26)	8.21	¿?	N/A	¿?	3.36
L (Lure)	High Supervision (N = 24)	8.95	8.95	0	N/A	3.31
	Low Supervisión (N = 24)	8.58	5.41	3.17 (37%)	12%	3.27
	No Supervision (N = 23)	8.87	¿?	N/A	¿?	3.20
TL (Triple Lure)	High Supervision (N = 22)	7.40	6.95	0*	N/A	3.23
	Low Supervisión (N = 22)	8.63	4.54	4.09 (47%)	0%	2.29
	No Supervision (N = 20)	8.80	¿?	N/A	¿?	2.11

Units Declared are the number of units the participants affirmed to finish. Units Actually Completed are the units participants really finished, checked by researchers in HS and LS condition (in NS it was not possible). Cheating is measured by the difference between Units Declared minus Units Actual Completed.

* Differences in TL High Supervision between Units Declared minus Units Actual Completed are due to mistakes, it is not a measure of cheating.

Using a time-proxy cheating measure (see [10]), in the same 3x2 ANOVA (now including NS), we found a main effect of both factors and interaction: Supervision [F(2,127) = 35.31; p <.001; η^2^ = .357], Presence of Others [F(2,127) = 204.64; p <.001; η^2^ = .617], and Interaction [F(2,127) = 31.12; p <.001; η^2^ = .329]. Time spent to complete the task in the FP situation was the same among the three Supervision conditions (all p>.99), while in I, participants spent more time in HS (3.23) than in LS (2.13; p<.001) or NS (2.13; p<.001). This is important because, as we have seen, people in LS cheated (declaring more units than they really did) and spent significantly less time than HS. As there was no significant difference between the time spent in the Isolated NS (2.13) and LS (2.13) conditions (p>.99) we can suspect that participants in NS cheated too. Finally, comparing LS and NS conditions, we found no significant differences on time spent or units declared for either FP or I situations, suggesting that the different types of “lack of supervision” (LS & NS) affected individual behavior in a similar manner.

In summary, accordingly to our Cheating, Performance, and Time results, in isolated situations under both Low and No Supervision people cheat. They declared more units than they really did (LS), and they spent less time doing the task than under High Supervision (both LS and NS). However, although dishonest behavior also appeared in FP as shown by cheating results, it was smaller than in I situations: we found a significant reduction in cheating and no differences between HS, LS, or NS conditions in time spent doing the task. This suggests that, although participants completed fewer units and declared more in LS (dishonest behavior), they were using the same amount of time than people in HS. While it is possible that workers invested more time to do each unit, it is much more likely that workers simply pretended to do the task under Low or No Supervision in the presence of Familiar Peers, with the intention to cheat. But, would these effects also appear when people suspect that others in the group may be cheating? What could happen if people suspect that someone may be cheating? We are showing interesting results investigating these questions in the next two experiments. In Experiment 2, we were interested in studying how a “suspiciously-fast participant” may affect group behavior using a confederate “Lure”. Could this lure change the norms of honest behavior for the group? Because participants were unaware of the other monitoring conditions, we hypothesized that the reaction to the Lure would vary by condition. We expected that participants in the HS condition would think that the confederate was particularly efficient, pressuring them to finish the task faster. In the LS and NS conditions, however, participants might believe that the confederate could be a cheater. If participants in these conditions wanted to win the social reward, we would expect that the lure’s actions increase participants’ “band of acceptable dishonesty”.

### Experiment 2: The Lure Effect

***“… If a single person breaks the norm and crosses*, *the temptation to jaywalk increases*. *Still*, *the action of the group holds sway*. . .*”***

## Method

### Participants

A total of 71 individuals (63% male; *M*_*age*_ = 20, *SD* = 1.6) participated in the study for approximately 3€. All participants were undergraduate students at a university in Madrid, Spain. The study took less than 30 minutes and was conducted with paper and pencil.

### Design and procedure

Experiment 2 was a replication of Experiment 1 only in the Familiar-Peer situation, but using a confederate: the “Lure”. That is, in the present experiment we have only one between-subjects factor, Supervision (HS, LS & NS). The Lure was randomly selected from the group and given instructions to hand in his or her results to the researcher exactly 10 minutes after starting the task. After those ten minutes, the confederate went to the researcher to hand in his or her work. The researcher observed all the units and specified to the confederate that it was correct. The number of units that the confederate finished was unknown to the rest of participants.

## Results and Discussion

### Performance

Mean number of units declared in all conditions for Experiment 2 is also reported in Table 1. A one-way ANOVA showed no differences between the three conditions of Supervision (F<1). To compare results of present experiment and previous experiment we conducted a 3x3 ANOVA with Supervision (HS, LS & NS) and Presence of Others (I, FP & L, as Lure). We found only a main effect of supervision [F(2,195) = 3,265; p = .04; η^2^ = .03]: the number of sheets declared in HS (*M* = 9.24) was significantly greater than in NS (*M* = 8.33; p = .043).

### Cheating

Again, mean cheating measures for each condition can be found in Table 1. In the T-test for Supervision we found significant differences between HS (*M* = 0) and LS (*M* = 3.17) conditions [t(46) = 9.35; p<.001; d = 2.7], showing that people were cheating again under low supervision (LS). Running a 2x3 Anova with Supervision (HS & LS) and Presence of Others (I, FP & L) including results from both Experiments 1 & 2, we found main effects of Supervision [F(1,132) = 277.99; p <.001; η^2^ = .68], Presence of Others [F(2,132) = 8.88; p = <.001; η^2^ = .12], and Interaction [F(2,132) = 10.70; p <. 001; η^2^ = .14]. Participants were cheating in all I, FP, and L conditions (as shown by the differences found between HS and LS; p<.001 in all comparisons), but cheating was larger for I (*Mean* Difference = 4.5) than for FP (*Mean* Difference = 2.2; p<.001) or L (*Mean* Difference = 3.1; p = .04). However, no differences were found between L and FP (p = .13), showing that the presence of the Lure does not change the level of dishonest behavior in experiment 2, although it is incremented in our sample (from 2.2 in FP to 3.1 in L).

Finally, using our time-proxy cheating measure, we found no differences among the three groups (F<1; see Table 1 for mean times in each condition). In the same 3x3 ANOVA done for Performance, comparing experiment 1 and 2 we found again, main effects of Supervision [F(2,195) = 29.75; p <.001; η^2^ = .23], Presence of Others [F(2,195) = 123.85; p <.001; η^2^ = .56], and Interaction [F(4,195) = 20.94; p <.001; η^2^ = .30]. Differences were present for LS only between I and FP, and I and L (p <.001 for both), but again no differences were found between FP and L (p >.99). The same result appeared for NS, with no differences in the pattern of results for LS and NS.

All together Performance, Cheating, and Time results show that effects in the present experiment are similar to those found for the FP situation in Experiment 1. The mere suspicion of a single person cheating is not enough to increase dishonest behavior of the group. Nevertheless, the lure results do suggest a trend. In the FP condition, 26% of participants were honest (units declared and units actually completed were exactly the same), whereas in L only 12% of participants were fully honest, and no one was fully honest in I (see Table 1). Those results together with the trend-increment found in dishonest behavior, L (3.1) compared to FP (2.2), suggest that a stronger lure may increase dishonest behavior. In the next experiment, we have examined if more confederates are a sufficient lure to push the group toward cheating.

### Experiment 3: The Followers Effect

***“…If more than one person decides to jaywalk*, *though*, *the peer pressure is enough that the whole group may cross*.*”***

## Method

### Participants

A total of 64 individuals (59% male; *M*_*age*_ = 20, *SD* = 1.4) participated in the study for approximately 3€. All participants were undergraduate students at a university in Madrid, Spain. The study took less than 30 minutes and was conducted with paper and pencil.

### Design and procedure

Experiment 3 replicated Experiment 2, but used three confederate Lures (Triple Lure: TL) instead of only one (L). The confederates were randomly selected from the group and instructed to finish the task at exactly 10 minutes (the “lure”) and 11 minutes (two “followers”) after the task began.

## Results and Discussion

### Performance

**Fig 1:**
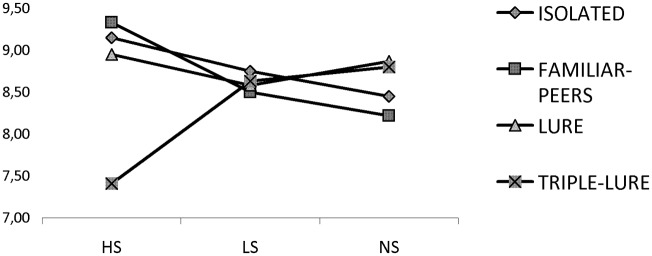
Number of sheets declared in the HS, LS, and NS conditions. Number of units reported in different supervision situations when participants are in isolated situations, or when the task is done individually but in the presence of other people (with or without confederates’ manipulations).

Again, mean number of units declared in all conditions is reported in Table 1. The one-way ANOVA on Supervision showed a significant main effect [F(2,61) = 5.21; p = .008; η^2^ = .15], contrary to results found in previous experiments. Specifically, differences were found between HS with both LS (p = .03) and NS (p = .01). That is, performance was reduced in HS compared to the other conditions. Moreover, it is the first condition among the three experiments where errors can be found while doing the task (see Table 1 for TL). Running a 3x4 ANOVA including all experiments with Supervision (HS, LS and NS) and Presence of Others (I, FP, L and TL) we only found a main effect of the Interaction [F(6,256) = 3.24; p = .004; η^2^ = .071]. The differences showed up only in HS between TL and the rest of the situations I (p = .002), FP (p <.001) and L (p = .005). As shown in Fig 1, for HS there is a significant reduction in units declared for TL, compared to the other situations (I, FP and L). In general, it seems that the presence of a Triple Lure affects performance under high supervision by reducing accuracy and increasing errors.

### Cheating

Mean cheating measures are reported once again in Table 1. The T-test for Supervision showed significant differences between HS (*M* = 0) and LS (*M* = 4.09) conditions [t(42) = 9.60; p = .003; d = 2.9]: like in previous experiments, people are cheating in LS. In a 2x4 ANOVA incorporating all the experiments with Supervision and Presence of Others as factors, we found main effects of both factors [F(1,174) = 365.85; p <.001; η^2^ = .68] and [F(3,174) = 9.40; p = <.001; η^2^ = .14], respectively, as well as a significant Interaction [F(3,174) = 7.10; p <. 001; η^2^ = .11]. Again, there is a difference between HS and LS for I, FP, L and TL (all p<.001), showing that participants cheated in all situations. Importantly, the level of cheating in TL is similar to I (p>.99), suggesting that the Familiar-Peer effect found in Experiments 1 and 2 has now disappeared in the Triple Lure situation.

**Fig 2:**
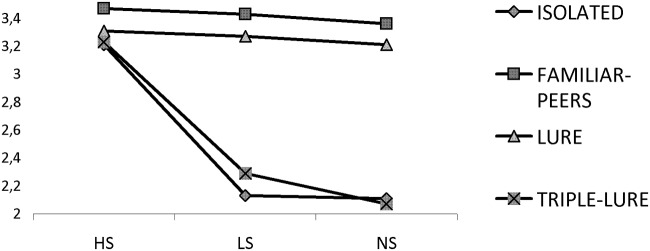
Time per units in the HS, LS, and NS conditions. Time spent in different supervision situations when participants are in isolated situations, or when the task is done individually but in the presence of other people (with or without confederates’ manipulations).

In the same vein, these results are very similar to those found in the analysis of the time-proxy cheating measure. We found a main effect in the one-way ANOVA for Supervision [F(2,61) = 149.90; p = <.001; η^2^ = .83] significant for all comparisons (p<.001) including differences between LS and NS (p = .03). It means that, in contrast to results found in previous experiments, participants in NS spent less time per declared unit than participants in LS, suggesting that seeing three lures increased the level of cheating when participants were 100% sure that they would not be caught (NS). Likewise, in the 3x4 ANOVA with Supervision and Presence of Others as factors, there was a main effect of both factors [F(2,256) = 88.27; p = <.001; η^2^ = .41] and [F(3,256) = 140.47; p = <.001; η^2^ = .62], respectively, and Interaction [F(6,256) = 23.98; p = <.001; η^2^ = .36]. There were no differences in HS conditions among Presence of Others situations (I, FP, L and TL; p = .245 for the biggest difference). But more importantly, the pattern of results is the same between I and TL situations, and between FP and L, as can be clearly seen in Fig 2 (just like the results found for Cheating). That is, when there is a clear suspicion of cheating (TL) under low supervision, dishonest behavior is increased, matching that of an isolated work environment. Moreover, “clear-cheating suspicion” (three lures) in NS makes a difference even with LS and I, likely because workers in the NS condition are very sure that the lures are clearly cheating.

### Efficiency Measurement

Participants’ efficiency could be measured directly only in the HS condition. In the NS condition, it is impossible to calculate efficiency because we cannot record the number of units actually completed. In the LS condition, we know the number of units actually completed as well as the time spent by participants doing the task. However, merely dividing time by real units assumes that participants worked efficiently throughout the task and only cheated at the very end of the session by stating a greater number of units declared than those truly completed. In reality, it may be likely that dishonest participants could spend time during the task simply doing nothing. That strategy would allow dishonest workers to reduce the likelihood of being caught (or at least decrease the suspicion of cheating) by the instructor or by their peers. To get a deeper understanding of how much time might be taken up in the LS and NS conditions dishonestly idling, we have calculated a time proxy for *efficient* work. The average time per unit in the HS condition (where participants could not cheat) is used as a proxy for the time it would take participants in the LS condition to actually finish a sheet. Multiplying this value by the number of real sheets finished in the LS condition, we obtain a proxy for the time that it should have taken a participant to finish the whole task. That is:

Efficiency_Time_Proxy=Number of sheets actually completed * Time per unit in the HS condition

**Fig 3:**
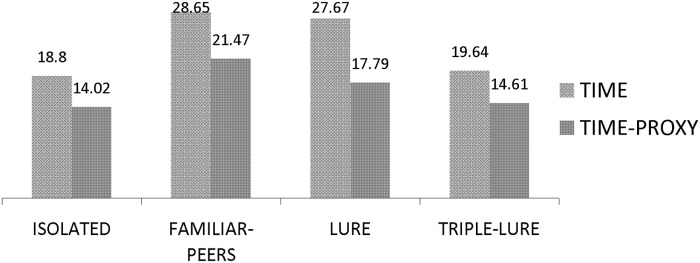
Efficiency in terms of time in the Ignored conditions. The average time per unit in the HS condition (where participants could not cheat) is used as a proxy for the time it would take participants to actually finish a sheet in the LS condition. Multiplying this value by the number of real sheets finished in the LS condition, we obtain a proxy for the time that it should have taken a participant to finish.

The difference between a participants´ real time spent and *efficiency time proxy* represents the time spent pretending to work. As we can see in Fig 3, the average difference in the four conditions (I, FP, L and TL) was 4.78, 7.18, 9.88 and 5.03 minutes, respectively. In a one-way ANOVA on those differences with Presence of Others as a factor, we found a main effect [F(3,88) = 6.69; p = .000; μ2 = .18], pointing out significant differences between L and I (p = .001), and between L and TL (p = .002). No differences were found between L and FP (p = .191). Together, these results show that in TL, participants not only cheated more (the proportion of finished sheets compared to those declared was the smallest), but they also spent more time pretending to work.

### Ethics Statement

Experiments were approved by the institutional review board of the Economics and Business Faculty of the Universidad Complutense de Madrid chaired by the Vice-Dean of Research and Postgraduate Studies. According to the institutional review board we did not use informed consent a priori. Informing participants that we were studying cheating would distort the results of the experiments. The data was analyzed anonymously. After completion of the experiments, participants were invited to a second phase of the experiment in which we explained the objective of our study and asked for the informed consent a posteriori. None of the participants expressed objection to use of their data.

## General Discussion

Peer effects are very important in organizations. Here, we show that working in the presence of others is an effective mechanism to constrain dishonest behavior and maintain similar levels of performance under low supervision work situations. Nevertheless, people’s behavior can also be modulated by competitive situations under pressure. If a subset of workers finishes a task unusually quickly to win a social reward, cheating of the whole group increases when supervision is low. With direct supervision, the same behavior causes the group’s overall performance to suffer.

With the same task structure and social rewards, participants working in a familiar-peer group have demonstrated equal performance compared to those working alone. But more importantly, those workers in familiar-peer groups cheat less than those working in isolated situations, according to findings of experiment 1. This suggests that the mere presence of others acts as an indirect supervision system for workers. When a task is completed in the presence of others, individuals can indirectly monitor their peers’ performance. McCabe, Trevino & Butterfield [24] hypothesized that cheating would be reduced when there is a stronger perception that cheaters can be caught. This is the case during indirect supervision by one’s peers, and it is particularly true when compared to the individual/unsupervised condition. Results found for cheating in experiment 1 are in accordance with this, and also for time spent doing the task under Familiar-Peer situations. When there is a risk of being labeled as a cheater, people prefer to keep their honest reputation intact by increasing honest behavior, also assuring that the time spent to do the task matches their peers. In terms of reputation-standing mentioned previously, it may be more important for a worker to keep their reputation of honesty over the long term than give it up at the expense of a short term increase in standing within his/her working-group.

But in a typical workspace things are usually more complicated. Work pressure given by rewards (social or economic) and others’ honest/dishonest behavior can significantly modify individual behavior. When a group of workers finishes a task unusually (and maybe suspiciously) quickly, cheating and performance can be modulated. We have observed two different effects on people’s behavior in experiment 3 depending on the level of supervision. Firstly, in a supervised setting where workers cannot cheat and believe that the rest of their peers are unable to cheat as well (HS conditions), when a social reward like prestige is included, the pressure to win the reward can cause workers to try to be faster, finish fewer units, and make more mistakes. Previous research has shown that very high economic reward can have a detrimental effect on performance [7]. Our results suggest that not only do high economic incentives decrease performance, but so do social incentives. This is contrary to results found by Falk & Ichinio [25] showing that peer effects raise the overall average productivity significantly. In other words, Falk & Ichinio [25] suggest that “bad apples,” far from damaging “good apples,” seem instead to gain in quality when paired. Nevertheless, there are three important differences between Falk’s work and ours. First, Falk and Ichinio did not run tasks in big groups; instead, their research was done using groups of two. Second, these two participants were complete strangers. Unlike our natural experiment, Falk’s participants would not be required to work together again after the experiment itself. Finally, Falk’s participants did not receive any incentives related to their performance. On the other hand, Falk and Ichinio’s findings do agree with our FP and L conditions in experiments 1 and 2, where familiar peer effects seem to increase honest behavior compared to the isolated task, even though participants had the opportunity to cheat just as much as in any other condition.

Secondly, in settings with less or no supervision, competition increases cheating. In a world where encounters with dishonesty are frequent, it is important to know if exposure to other people’s unethical behavior can increase or decrease someone else’s cheating [1]. Carrel, Malmstrom, & West [26] modeled how the addition of one cheater to a group “creates” roughly three new cheaters. Our results show that, when workers think that others can be cheating, the amount of cheating increases.

It is also important to highlight the difference between knowing when a group member is a cheater versus when there is only suspicion. With one confederate, we did not find a widespread increase in cheating (experiment 2). In this case, the lure was a suspected cheater, but participants had no way to confirm their suspicions. Gino et al. [1] found that when a confederate belonged to the group and cheated ostentatiously by finishing a task impossibly quickly and leaving the room with the maximum reward, participants’ level of unethical behavior increased. In Gino´s research, participants were completely sure about the existence of cheating. However, in a shared workplace, it is common for workers to observe potential dishonesty from others without confirmation. Because co-workers must continue to interact with each other on a regular basis, blatant dishonesty is usually frowned upon. As such, workers are careful to hide their dishonesty as a social norm. Then, like in our study, co-workers may suspect dishonest behavior of their colleagues, but will not be able to confirm it. That is probably why we find only a tendency of the group to increase cheating in experiment 2, whereas cheating increases significantly in experiment 3 when suspicion increases.

In summary, if workers have the opportunity to cheat, they will cheat. The amount of cheating increases with social incentives, such as standing. Importantly, the effects of incentives on dishonesty can be completely negated by the mere presence of a familiar peer group, suggesting that reputation is more important than standing. The advantage of working in familiar peer groups, however, is not guaranteed. When the risk of losing reputation decreases (for example when you are aware of the presence of other colleagues cheating), people will again increase their level of cheating. Moreover, when people cannot cheat under high levels of supervision and others in their familiar peer group work substantially faster to win prestige, pressure affects workers’ performance negatively. The existence of familiar peer effects found in this article raises interesting questions concerning efficient design of the workplace. Should employees work alone in an individual room or share the common space? If they do work with others, how should be workers optimally grouped to assure that competition is constructive? Our work suggests that familiar-peer supervision may be an effective way to encourage honesty.

## Supporting Information

S1 Table Database. Database in the HS, LS, and NS conditions and gender.
(XLS)
